# When to Consider a Diagnosis of Maturity-Onset Diabetes of the Young: Precise Diagnosis Leads to Better Management and Quality of Life for the Patients

**DOI:** 10.1055/s-0045-1802584

**Published:** 2025-02-11

**Authors:** Yujia Gao, Kalyan Mansukhbhai Shekhda, Sarah N. Ali

**Affiliations:** 1 UCL Medical School, University College London, London, United Kingdom; 2 Department of Diabetes and Endocrinology, Royal Free London NHS Foundation Trust, Pond St, London, United Kingdom

**Keywords:** MODY, genetic diabetes, *HNF1A*, *HNF4A*, *GCK-MODY*

## Abstract

Maturity-onset diabetes of the young (MODY) is often misclassified and can significantly impact the management of these patients and their families. We present three cases initially diagnosed as type 1 diabetes mellitus (T1DM), type 2 diabetes mellitus (T2DM), and fasting hyperglycemia, which were later identified as MODY. A 38-year-old Caucasian lady, previously diagnosed with T2DM, was referred to the diabetes antenatal clinic. She was treated with gliclazide and metformin before pregnancy. She required insulin glargine during her pregnancy. Her diabetes autoantibodies were negative. MODY was suspected and genetic testing confirmed *HNF1A* MODY gene mutation. A 57-year-old Caucasian lady was diagnosed with T1DM at the age of 18 years. Since diagnosis, she was treated with insulin glargine without any short-acting insulin, yet persistently suffered from hypoglycemia. MODY was suspected and genetic testing confirmed *HNF4A gene* mutation. A 33-year-old South Asian lady was referred to a diabetes clinic for suspected T2DM due to strong family history of T2DM, ethnicity, and persistently elevated fasting blood glucose levels. Her genetic testing confirmed *GCK* - *MODY* ( *Glucokinae-maturity-onset diabetes of the young* ). MODY represents a group of genetic diabetes that can often go unrecognized due to misdiagnosis. Achieving an accurate diagnosis is important as it guides appropriate treatment strategies, improves patient outcomes, and has an impact on other family members due to the hereditary nature of the condition. Employing a systematic approach is crucial. Our cases highlight that it is never too late to challenge the diabetes classification.

## Introduction

Maturity-onset diabetes of the young (MODY) represents a group of monogenic diabetes caused by autosomal dominant inherited defects in gene related to insulin secretion and estimated to account for 1 to 5% of the diabetes population. 1 MODY appears to primarily affect the Caucasian population, with an onset of diabetes typically occurring before the age of 30 years. 1 The scarcity of prevalence data for MODY in non-Caucasian ethnicities is conspicuous, likely stemming from an insufficient depth of research and genetic investigations in these ethnic groups. MODY exhibits clinical characteristics distinct from type 1 diabetes mellitus (T1DM) and type 2 diabetes mellitus (T2DM) such as multigenerational family history of diabetes, absence of pancreatic beta cell autoimmunity (present in T1DM), and insulin resistance (present in T2DM). 2

The current method for detecting MODY requires clinical recognition by a medical professional, use of the *Exeter MODY calculator* , and subsequent genetic testing. 2 3 However, about 80% of MODY cases receive an inaccurate initial diagnosis 1 due to lack of clinical recognition or understanding among general physicians, compounded by overlapping clinical presentations with T1DM and T2DM. 4 Adults not needing immediate insulin treatment are often labeled as T2DM, whereas those who are on insulin treatment since the diagnoses are labeled as T1DM. 1 Upon revisiting their diagnoses, it may be revealed that these individuals actually have MODY. Additionally, the increasing prevalence of obesity has introduced further complexity to these distinctions. Obesity, which is often associated with insulin resistance, has blurred the lines between these diabetes subtypes, as insulin resistance is now commonly observed in individuals with both T1DM and MODY. 5 Accurate molecular diagnosis therefore holds significant implications for patient management and long-term outcomes, guiding appropriate treatment strategies and influencing familial diagnoses. For example, some patients may be able to come off insulin, or if diagnosis was GCK, then no treatment will be needed and genetic testing for offspring or other family members would be warranted. In this context, we present the cases of three patients of MODY from different ethnicities who were misdiagnosed as T2DM, T1DM, fasting hyperglycemia likely T2DM.

## Case Presentations

### Case 1

A 38-year-old Lithuanian lady was referred to the diabetes antenatal clinic at 8 weeks of her first pregnancy with glycosylated hemoglobin A1c (HbA1c) level of 52 mmol/mol (6.9%). She was previously diagnosed with T2DM at the age of 25 years on a routine testing and had been managing well on gliclazide 40 mg once daily, metformin 1 g twice daily, and sitagliptin 100 mg once daily preconception. The patient maintained a lean physique with a body mass index (BMI) of approximately 20 kg/m ^2^ and had no known family history of diabetes or renal diseases. Early in her pregnancy, she was kept solely on metformin 1 g twice daily and she experienced intermittent fasting hyperglycemia. At 16 weeks of gestation, she was initiated on insulin glargine 2 units once daily. Due to her clinical features being distinct from T2DM, MODY was suspected. Screening tests for pancreatic autoantibodies, including glutamic acid decarboxylase (GAD) antibodies, islet antigen 2 (IA-2) antibodies, and zinc transporter 8 (ZnT8) antibodies, were negative. Subsequently, she was referred for the MODY genomic testing postpartum. The genomic testing results confirmed the presence of an *HNF1A MODY* gene mutation. She was continued on metformin and insulin therapy during pregnancy. During the postnatal period, adjustments were made to her treatment plans, which involved gradual weaning and discontinuation of insulin and addition of gliclazide. Her glycemic control is stable on oral hypoglycemic agents and no daily insulin requirements, with HbA1c of 45 mmol/mol (6.3%). Her child will need to be considered for genetic testing due to the 50% chance of inheriting the *HNF1A gene* mutation and therefore the development of diabetes.

### Case 2

The second case was a 57-year-old Caucasian lady who was diagnosed with T1DM at the age of 18 years. Her BMI has remained between 18 and 20 kg/m ^2^ . Since the diagnosis of her T1DM, she remained only on a small dose of daily basal insulin glargine 6 units daily without the need for any short-acting insulin. Despite this, she suffered from recurrent episodes of hypoglycemia over the years. She did not have any other medical condition, including Addison's disease. Her family history was unknown. A genetic cause of her diabetes was suspected based on clinical and biochemical features. Genetic testing confirmed *HNF4A gene* mutation. She was switched to gliclazide 40 mg once daily with good glycemic control and no further episodes of hypoglycemia. She has a son and grandchildren who do not have diabetes, but they will need to undergo genetic testing.

### Case 3

A 33-year-old lady of South-Asian ethnicity was referred to a diabetes clinic with persistent fasting hyperglycemia since the age of 24 years. Her fasting blood glucose levels remained between 5.5 and 6.4 mmol/L (99 and 115.2 mg/dL) since diagnosis. Despite losing significant weight and BMI from 21.5 to 19.9 kg/m ^2^ , her HbA1c remained 41 mmol/mol (5.9%). She had a strong family history of diabetes, with her father diagnosed with T2DM and sister suspected of having diabetes. Her diabetes antibody testing was negative. The diagnosis of *GCK-MODY* ( *Glucokinase-maturity-onset diabetes of young* ) was suspected, and genetic testing confirmed *GCK-MODY* genetic mutation.

## Discussion

MODY is an autosomal dominant group of genetic diabetes characterized by defective insulin secretary function of beta cell of pancreas 1 and exhibits clinical characteristics distinct from both T1DM and T2DM. 2 Key differentiators include lower HbA1c levels and a robust family history of diabetes at diagnosis in comparison to T1DM. In contrast to T2DM, MODY is characterized by a lower BMI, earlier diagnosis, lower glycated HbA1c levels at diagnosis, and diminished responsiveness to oral antidiabetic medications. 3 To date, at least 14 pathogenic mutations have been reported, with *HNF1A* (52%), *GCK-MODY* (32%), *HNF4A* (10%), and *HNF1B* (6%) being the most common in the United Kingdom. 1 Among these, some subtypes are distinguished by stable blood glucose levels, others by progressively worsening insulin secretion and glucose control, and the rest by extra-pancreatic characteristics such as macrosomia, renal cysts, and azoospermia. 6 We describe the differences in the phenotypic features of T1DM, T2DM, and common types of MODY in Table 1 .

**Table 1:** Difference between phenotypic features of T1DM, T2DM, and common variant of MODY

	*HNF1A* MODY (MODY3)	*HNF4A MODY* (MODY1)	*HNF1B* MODY (MODY5)	*GCK-MODY* (MODY2)	T1DM	T2DM
Age at onset (y)	< 25	< 18	< 25	Preadolescence	< 30	> 25
Disease manifestation	Progressive beta cell dysfunction	Progressive beta cell dysfunction	Beta cell development defect	Stable beta cell dysfunction Stable, mildly raised glucose	Progressive autoimmune loss of beta cell function	Insulin resistance, very slowly progressive beta cell dysfunction
First-degree relative with diabetes	50–60%	50–60%	50%	Often undiagnosed or reported as IGT/GDM	2–4%	50%
Insulin resistance	No a	No a	No a	No a	No a	Yes
Islet antibodies	Negative	Negative	Negative	Negative	Mostly positive early at diagnosis	Negative
Risk of microvascular complications	High	High	High	Rarely	High	High
Extra-pancreatic features	Renal glycosuria	Macrosomia with hyperinsulinemic hypoglycemia; low levels of apolipoproteins and triglycerides; neonatal macrosomia; neonatal hypoglycemic events	Genitourinary malformations (renal cysts, azoospermia, uterus anomaly)	Not described	Other autoimmune diseases, e.g., autoimmune hypothyroidism, vitiligo	Acanthosis nigricans, obesity, vascular disorders
Treatment	Insulin secretagogues (sulfonylureas, meglitinides), GLP-1 RA, SGLT-2i, insulin	Diet, sulfonylureas, insulin	OHAs (sulfonylureas or repaglinide), insulin	Treatment is usually not necessary	Insulin	Diet, metformin, DPP-4 inhibitors, GLP-1 RA, OHAs, SGLT-2i, insulin

**Abbreviations:** DPP-4, dipeptidyl peptidase 4; GCK, glucokinase; GDM, gestational diabetes mellitus; GLP-1 RA, glucagon-like peptide-1 receptor agonist; HNF1A, hepatocyte nuclear factor 1-A; HNF1B, hepatocyte nuclear factor 1B; HNF4A, hepatocyte nuclear factor 4A; IGT, impaired glucose tolerance; MODY, maturity-onset of diabetes of the young; OHA, oral hypoglycemic agent; SGLT-2i, sodium-glucose co-transporter-2 inhibitor; T1DM, type 1 diabetes mellitus; T2DM, type 2 diabetes mellitus.

a Insulin resistance can occur with comorbid obesity.

**Fig. 1:**
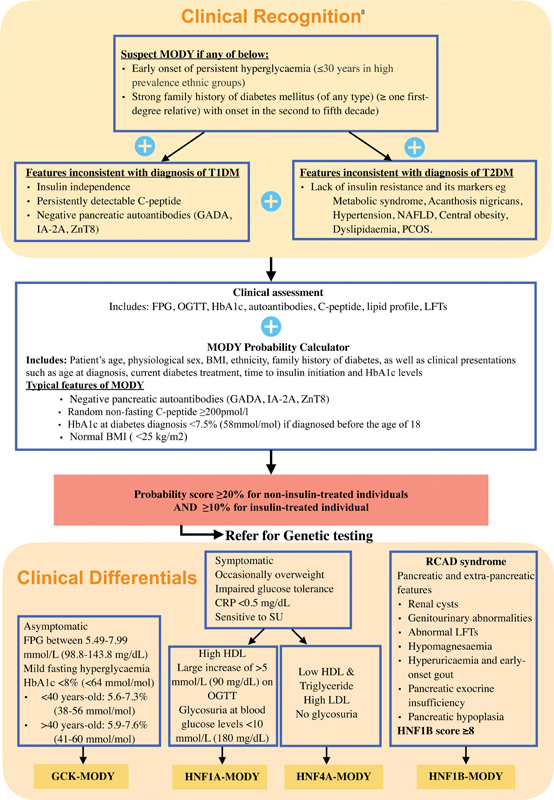
Clinical algorithm for recognition, assessment, referral consideration, and confirmation of MODY. BMI, body mass index; FPG, fasting plasma glucose; GADA, glutamic acid decarboxylase antibodies; HbA1c, glycated hemoglobin A1c; HDL, high-density lipoprotein; IA-2A, islet antigen 2 antibodies; LDL, low-density lipoprotein; LFT, liver function tests; MODY, maturity-onset of diabetes of the young; NAFLD, nonalcoholic fatty liver disease; OGTT, oral glucose tolerance tests; PCOS, polycystic ovarian syndrome; RCAD, renal cysts and diabetes syndrome; SU, sulfonylurea; T1DM, type 1 diabetes mellitus; T2DM, type 2 diabetes mellitus; ZnT8, zinc transporter 8 (ZnT8) antibodies. (Reference: https://www.mydiabetes.com/healthcare-professionals/mody-protocol/ )

The current method for detecting MODY requires clinical recognition by a medical professional, use of the *Exeter MODY calculator* , 3 and subsequent genetic testing. The main barriers to genetic testing for monogenic diabetes are often misdiagnosis due to insidious onset of symptoms, overlap of clinical features with T1DM and T2DM, and lack of clinical recognition or lack of awareness of MODY among clinicians. 4 In our patients, although patient in case 1 did not exhibit typical features of T2DM (e.g., obesity, signs of insulin resistance, older age of diagnosis), she was labeled as having T2DM. On the other hand, the patient in case 2, although she required very small amount of long-acting insulin with frequent hypos, she was labeled as having T1DM. Likewise, many times, adults who do not need immediate insulin treatment are often labeled as T2DM patients, whereas patients who are on insulin treatment since the diagnoses are labeled as T1DM patients. 1 However, these patients can have MODY, and about 80% of those with MODY will receive an inaccurate diagnosis at presentation. 1 The MODY probability calculator can aid in identifying individuals for genetic testing, at improved sensitivity and specificity compared to standard criteria. 3 A probability score of ≥20% for non-insulin-treated individuals and ≥10% for insulin-treated individuals indicates the necessity for genetic testing as per the National Health Services (NHS) England guidelines. 7 Making an accurate molecular diagnosis can significantly impact the management of these patients and the long-term outcome. 2 A flowchart for the diagnoses of MODY is presented in Fig. 1 . Although the prevalence of MODY in the non-Caucasian population is low, this could be due to lack of the following: awareness among treating physicians, testing for MODY genetic testing, and large population-based genetic studies.

The management of MODY is contingent upon the presence of specific genetic abnormalities. 6 Molecular diagnosis plays a pivotal role in determining the optimal treatment course for the majority of MODY patients. 6 Patients with mild hyperglycemia at diagnosis may effectively manage their condition through dietary adjustments. In the case of GCK-MODY, characterized by mild, stable fasting hyperglycemia (HbA1c range of 5.6 - 7.6% (37.7 - 59.6 mmol/mol)), antihyperglycemic therapy is generally unnecessary, given the absence of significant differences in HbA1c levels or micro- and macrovascular complications between treated and untreated GCK-MODY individuals, 8 unless coexisting comorbidities such as T1DM or T2DM, obesity, and pregnancy are present. 9 Individuals with *HNF1A* and *HNF4A* MODY typically exhibit high sensitivity to sulfonylureas like gliclazide. 6 Initiation of sulfonylureas should be gradual, with dosage titration to achieve the desired effect. An alternative to sulfonylureas is glucagon-like peptide-1 receptor agonist (GLP-1RA), such as dulaglutide, semaglutide, and liraglutide, which are glucose dependent and possess broad efficacy. 10 While knowledge of GLP-1RA use in MODY is currently limited, these agents have demonstrated potential in reducing hypoglycemia, improving cardiovascular risk factors, and holding promise for specific MODY phenotypes like *HNF1A-MODY* . 10 Although there are reports of use of SGLT2i in patients with *HNF1A-MODY* , the use of SGLT2i is associated with increase in incidence of glycosuria and subsequently poses an increased risk of euglycemic diabetic ketoacidosis (DKA) and genital infection due to reduced endogenous insulin secretion characteristic of MODY patients. 11 12 These considerations emphasize the need for careful patient selection, close monitoring, and further studies to evaluate the risk–benefit profile of oral hypoglycemic agents in MODY patients.

In conclusion, achieving an accurate diabetes diagnosis is important as it guides appropriate treatment strategies, improves patient outcomes, and impacts diagnosis for family members. Employing a systematic approach involving good clinical history, diabetic autoantibodies testing, and calculating the probability using the *Exeter MODY calculator* is crucial. Our documented cases underscore the enduring relevance of the challenges of the prevailing diabetes classifications, emphasizing that it is never too late to embark on the quest for diagnostic precision.
